# Targeting NLRP3 Inflammasomes in Myocarditis: Potential Therapeutic Strategies and Clinical Translation

**DOI:** 10.1155/sci5/6560386

**Published:** 2026-05-19

**Authors:** Faizah D. Retnowati, Dunia R. Halawa, Zaid H. Maayah, Atiyeh M. Abdallah

**Affiliations:** ^1^ Department of Biomedical Sciences, College of Health Sciences, QU Health Sector, Qatar University, Doha, Qatar, qu.edu.qa; ^2^ Department of Pharmaceutical Sciences, College of Pharmacy, QU Health Sector, Qatar University, Doha, Qatar, qu.edu.qa

**Keywords:** autoimmune, inflammation, myocarditis, NLRP3 inflammasome, treatment

## Abstract

Myocarditis can be caused by viral infections, systematic autoimmune diseases, and as a reaction to prescribed medications, and the nucleotide‐binding domain leucine‐rich repeat pyrin domain‐containing 3 (NLRP3) inflammasome is implicated in its pathophysiology. Here, we explore pharmaceutical and other therapies that modulate NLRP3 activation and may therefore be useful for treating myocarditis. Pharmaceutical agents such as colchicine, SGLT2 inhibitors (empagliflozin and canagliflozin), calpain inhibitors, and sacubitril/valsartan have shown promise in reducing inflammation and preserving cardiac function. Natural compounds, including morroniside and crocin, exhibit anti‐inflammatory and cardioprotective effects by targeting the NLRP3 inflammasome. While these treatments have shown preclinical potential, successful clinical translation of inflammasome‐targeting drugs will require efforts to overcome recruitment challenges, flawed trial designs, high dropout rates, safety and toxicity concerns, and a lack of biomarkers. Natural products may offer a practical solution to these issues, providing safer therapeutic options and providing innovative approaches for managing myocarditis and preventing long‐term complications.

**Trial Registration:** ClinicalTrials.gov identifier: NCT05855746

## 1. Introduction

Myocarditis describes the inflammation of the heart muscle, and it incurs a significant health burden worldwide; it is also the most common cause of sudden cardiac death. The epidemiology of myocarditis is complex, with data from the Global Burden of Disease study indicating that, between 1990 and 2017, its incidence grew by 59.6% and deaths from myocarditis increased by 71.4% [1]. The prevalence of myocarditis increases with age in both men and women, although it is more common in men before the age of 80–84 [2], reversing thereafter [2]. Myocarditis has multiple etiologies, including viral infections, systematic autoimmune diseases, or drugs such as immune checkpoint inhibitors (ICIs) used in cancer management [3].

The nucleotide‐binding domain leucine‐rich repeat pyrin domain‐containing 3 (NLRP3) inflammasome is directly implicated in the inflammatory responses seen in myocarditis [4–6]. Inflammasomes are multiprotein complexes that play a role in inflammatory signaling and the innate immune system, releasing proinflammatory cytokines such as interleukin (IL)‐1β and IL‐18 [4]. Inflammasome complexes have three key components: a sensor, a caspase‐recruitment domain (CARD), and inflammatory caspases. Activation follows a two‐step process involving the transcriptional upregulation of the sensor component, which is essential for inflammasome activation, and upregulation of IL‐1β, a downstream effector cytokine produced by inflammasomes [4].

The NLRP3 inflammasome is primarily expressed in myeloid cells such as macrophages and dendritic cells. They respond to bacterial, fungal, and viral stimuli, as well as damage‐associated molecular patterns (DAMPs)—including extracellular ATP and cholesterol—through association with absent in melanoma 2 (AIM2) [7]. AIM2 is a key initiating protein in the inflammasome family, acting as an immune receptor that recognizes a specific molecular pattern, double‐stranded DNA (dsDNA), released during cell damage or pathogenic attack. NLRP3 activation involves priming through pattern recognition receptors (PRRs), leading to the recruitment of the apoptosis‐associated speck‐like protein containing a CARD (ASC) and activation of caspase‐1 [4, 7]. This process results in cleavage of IL‐1β and IL‐18, triggering inflammatory responses, activation of gasdermin‐D, and eventually pyroptosis [8]. Through this mechanism, NLRP3 plays a crucial role in regulating inflammation in various conditions, including myocarditis.

Other than myocarditis, the NLRP3 inflammasome plays a role in several other cardiovascular diseases, including atherosclerosis, myocardial infarction, diabetic cardiomyopathy, and heart failure. In these conditions, the activation of the NLRP3 inflammasome contributes to inflammation, fibrosis, adverse cardiac remodeling, and impaired cardiac function through the release of IL‐1β and IL‐18 [4]. The Canakinumab Anti‐Inflammatory Thrombosis Outcomes Study (CANTOS) trial further demonstrated the importance of inflammasome‐related inflammation in cardiovascular disease, in which IL‐1β inhibition was associated with a reduction in recurrent cardiovascular disease events [9]. Overall, these findings highlight the broader role of the NLRP3 inflammasome in cardiovascular pathology and support targeting this pathway in myocarditis.

**FIGURE 1:**
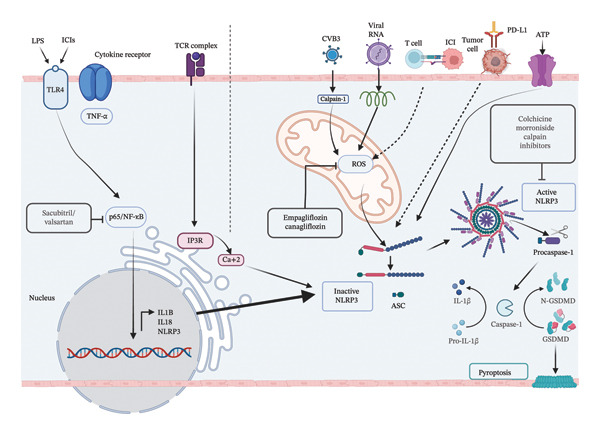
Involvement of NLRP3 inflammasomes in inflammatory responses in myocarditis, cardiac remodeling, and myocardial dysfunction.

NLRP3 inflammasome‐mediated inflammation in myocarditis ultimately results in cardiac remodeling and myocardial dysfunction, the most common outcomes of this disease (Figure 1) [10]. Several therapeutic strategies proposed for myocarditis inhibit the activation of the NLRP3 inflammasome. This review summarizes current knowledge on the pharmaceutical and natural treatments used to treat viral, autoimmune, and ICI‐related myocarditis by targeting the NLRP3 inflammasome. By examining these strategies, the review aims to highlight potential interventions that might be useful for mitigating the impact of inflammasome activation in myocarditis and improve clinical outcomes.

## 2. Targeting Inflammasomes to Manage Myocarditis

Efforts to develop therapeutic strategies targeting the NLRP3 inflammasome over recent years have provided new approaches for treating myocarditis, with each strategy exploiting a different mechanism in different clinical applications (Table 1) [11–18]. Targeting the NLRP3 inflammasome is an attractive approach for myocarditis, as its activation is closely linked to inflammation‐driven processes and outcomes, making it a promising target for mitigating disease progression and improving patient outcomes [6].

**TABLE 1:** Treatment of myocarditis by targeting NLRP3 inflammasomes.

Type of myocarditis	Treatment	Study model	Effect	Reference
Viral	Colchicine 5 μmol/kg for 7 days orally	C57BL6/j mice injected with CVB3	↓ NLRP3	[11]
			↓ ASC	
			↓ Caspase‐1	
			↓ IL‐1β	
			↓ Fibrotic markers	
	Overexpression of calpastatin (calpain inhibitor)	Calpastatin transgenic mouse strain (TgCAST) injected with CBV3	↓ NLRP3	[12]
			↓ Cleaved caspase‐1	
			↓ Cleaved caspase‐11	
			↓ GSDMD	
			↓ IL‐1β	
	Morroniside 20 mg/kg via IP injection	Wild‐type Sprague‐Dawley (SD) male rats injected with CBV3	↓ NLRP3	[13]
			↓ IL‐1β	
			↓ IL‐18	
			↓ Fibrosis	
			↓ Apoptosis	
			↑ LV function	

Autoimmune	Empagliflozin 30 mg/kg/day orally for 14 days	BALB/c mice injected with α‐myosin heavy chain peptide	↓ Inflammatory cell infiltration	[14]
			↓ Fibrosis	
			↓ Collagen deposition	
			↓ STAT3 pathway activation	
			↓ M1 macrophage polarization	
			↑ M2 macrophage polarization	
			↓ SGLT2	
	Canagliflozin 30 mg/kg/day orally for 21 days		↓ NLRP 3	[15]
			↓ IL‐1β	
			↓ IL‐18	
			↓ cTnT	
			↓ Th17 cell infiltration	
			↓ Apoptosis markers	
			↓ Myocardial damage	
	Sacubitril/Valsartan (Sac/Val) 20 mg/kg/day for 21 days		↓ Th17 cell differentiation	[16]
			↓ NF‐κB p65 signaling	
			↓ Inflammatory response	

Immune checkpoint inhibitors	Crocin	BALB/c mice treated with cardiac troponin I peptide and anti‐PD‐1	↓ NLRP3	[17]
			↓ Pyroptosis markers	
			↓ NF‐κB p65 pathway activation	
			↑ LV function	
			↓ Fibrotic markers	
	Empagliflozin 500 + 100 nM ipilimumab for 12 h	Ipilimumab‐treated MCF‐7 human breast cancer cells and AC16 human cardiomyocytes under hyperglycemia	↓ NLRP3	[18]
			↓ MyD88	
			↓ IL‐1β	
			↓ IL‐6	
			↓ Cardiotoxicity	
			↑ Anticancer efficacy	

### 2.1. Pharmaceutical and Other Treatments That Treat Viral Myocarditis by Targeting NLRP3

Viral myocarditis, predominantly caused by coxsackievirus B3 (CVB3), is a significant cause of inflammatory cardiomyopathy, leading to progressive cardiac injury and heart failure [6]. The disease progresses through three distinct phases: (i) an acute phase (1–7 days), marked by viral entry into cardiac cells and activation of the innate immune system; (ii) a subacute phase (1–4 weeks), dominated by the adaptive immune response; and, if unresolved, (iii) a chronic phase lasting months to years, characterized by persistent inflammation, immune‐mediated damage, and cardiac remodeling [6]. Numerous studies have shown the importance of the NLRP3 inflammasome in mediating inflammation and myocardial damage due to this infection [6, 19].

Several therapeutic strategies, including the use of medications and natural treatments, have the potential to target the NLRP3 inflammasome in viral myocarditis. Colchicine, a widely used anti‐inflammatory drug, has been shown to prevent the progression of viral myocarditis by directly modulating the NLRP3 inflammasome [11].

Colchicine has been extensively evaluated in human cardiovascular disease, particularly in atherosclerotic disease. Trials such as the COLchicine Cardiovascular Outcomes Trial (COLCOT) and the Low‐Dose Colchicine 2 Trial (LoDoCo2) reported reductions in inflammatory cardiovascular events. However, the results for different cardiovascular conditions have been heterogenous [20, 21]. For example, the COLCOT trial showed that low‐dose colchicine reduced cardiovascular events after myocardial infarction [21]. In contrast, results for acute coronary syndrome have been less consistent. Even though there was a decrease in cardiovascular events in the colchicine group, the Colchicine in Patients with Acute Coronary Syndromes (COPS) trial in patients hospitalized with acute coronary syndrome did not show a statistically significant reduction in major cardiovascular events [22]. Variable pathophysiology, as well as different dosing and timing of colchicine administration, may contribute to mixed findings, and the role of colchicine in myocarditis is far from certain.

Despite these mixed clinical findings, several preclinical studies have demonstrated the beneficial effects of colchicine in experimental models of myocarditis. In six‐week‐old mice injected with CVB3 to induce myocarditis, colchicine dosed at 5 μmol/kg body weight orally reduced the activation of NLRP3 inflammasome components and associated proteins such as ASC, caspase‐1, and IL‐1β in cardiac and splenic macrophages [11]. This suppression of inflammasome components preserved myocardial function and prevented excessive fibrosis by reducing the expression of fibrosis‐related genes and collagen deposition [11]. Furthermore, cardiac output was more efficient in colchicine‐treated mice compared with controls through a reduction in left ventricular (LV) interstitial fibrosis [11]. Fibrotic damage usually occurs due to CVB3‐mediated inflammasome activation, causing myofibroblast activation and consequent tissue fibrosis [11]. Importantly, colchicine treatment did not increase CVB3 viral load in treated mice, indicating the preservation of the host viral defense response [11]. Colchicine may therefore be useful in cases even where the virus persists, which is clinically important [11]. Overall, these findings suggest that colchicine is a promising therapy for viral myocarditis, effectively reducing NLRP3 inflammasome activation, preserving myocardial function, and preventing fibrosis without compromising the host’s antiviral response.

CVB3 also activates calpain, a protease involved in inflammation and cell death, in mice, injuring cardiac tissue by amplifying both canonical (NLRP3/caspase‐1) and noncanonical (caspase‐11) pyroptosis pathways [12]. Inhibition of calpain experimentally using transgenic mice overexpressing calpastatin, a natural calpain inhibitor, suppressed the activation of the NLRP3 inflammasome during infection with CVB3 to induce myocarditis [12]. This inhibition decreased disease severity, as demonstrated by a higher survival rate in the treatment group [12]. This positive therapeutic effect was attributed to the cardioprotective effects of the inhibitor, which promoted a decrease in serum cardiac troponin I (cTnI), an indicator of myocardial necrosis, reduced viral load, and decreased fibrotic tissue in treated mice [12]. Immunohistochemical analysis of heart tissue confirmed a reduction in pyroptosis markers, including caspase‐1, caspase‐11, and GSDMD, together with a reduction in IL‐1β, contributing to the therapeutic effects of calpastatin [12]. Collectively, these findings highlight the therapeutic potential of calpain inhibition in viral myocarditis, demonstrating its ability to reduce NLRP3 inflammasome activation, limit pyroptosis‐driven cardiac injury, and improve survival outcomes through enhanced cardioprotection.

Morroniside, a natural compound originating from *Cornus officinalis* (the Japanese cornelian cherry), has shown potential in reducing myocardial damage induced by CVB3 [13]. To test the potential cardioprotective effects of morroniside, male Sprague‐Dawley rats were injected with 20 mg/kg morroniside intraperitoneally and infected with CBV3 to induce myocarditis [13]. Heart function significantly improved in the treatment group, as demonstrated by improved heart rate, arterial pressure, and LV systolic pressure [13]. Mechanistically, morroniside inhibited the activation of the NLRP3 inflammasome, which in turn reduced the production of proinflammatory cytokines such as IL‐1β and IL‐18 [13]. This inhibition mitigated inflammation and myocardial cell apoptosis, thereby improving cardiac function and reducing tissue damage in rat models [13]. Histopathological analyses confirmed the protective effects of morroniside [13] through a reduction in apoptosis in heart tissues, highlighting its role in preserving heart tissue integrity and function during viral myocarditis. Overall, these results demonstrate the therapeutic potential of morroniside in viral myocarditis through the inhibition of NLRP3 inflammasome activation, reduced inflammation and apoptosis, and preserved cardiac function and tissue integrity.

Overall, therapeutic targeting of the NLRP3 inflammasome represents a promising route for managing viral myocarditis. Colchicine, calpain inhibitors, and morroniside mediate distinct mechanisms that reduce inflammation, prevent pyroptosis, and preserve myocardial integrity. These interventions not only attenuate the activation of the NLRP3 inflammasome but also improve systemic immune responses and myocardial remodeling. Future clinical trials are warranted to evaluate their efficacy and safety in human patients with viral myocarditis. However, it is worth noting that while several pharmaceutical agents targeting inflammatory pathways have progressed to clinical trials in patients with cardiovascular disease, natural compounds that modulate NLRP3 activation remain limited to preclinical studies. This gap highlights the need for further translational research to evaluate the clinical potential of naturally derived inflammasome inhibitors.

### 2.2. Pharmaceutical and Other Treatments to Treat Autoimmune Myocarditis by Targeting the NLRP3 Inflammasome

Autoimmune myocarditis is a serious inflammatory heart condition that can lead to dilated cardiomyopathy (DCM) and heart failure due to immune‐mediated damage [23]. Chronic myocarditis can arise through an autoimmune mechanism, where the body’s immune system mistakenly attacks cardiac tissues, either as a primary condition or as a secondary response to viral infections [23]. Therefore, myocarditis can form part of the clinical presentation of systemic autoimmune diseases such as systemic lupus erythematosus (SLE) [23]. One promising area of investigation to mitigate the immune‐mediated damage associated with autoimmune disease involves targeting the NLRP3 inflammasome.

Empagliflozin, a sodium/glucose cotransporter 2 (SGLT2) inhibitor known for its hypoglycemic anti‐inflammatory and antioxidant effects, has shown significant efficacy in reducing inflammation and activation of the NLRP3 inflammasome in various models of cardiovascular diseases [24, 25]. The potential therapeutic effect of empagliflozin in autoimmune myocarditis has been investigated in a mouse model of experimental autoimmune myocarditis (EAM) treated with empagliflozin orally (30 mg/kg/day) [14]. The results of this study demonstrated that, in empagliflozin‐treated mice, there was significantly less inflammatory cell infiltration, fibrosis, and collagen deposition in myocardial tissue, confirming its cardioprotective effect [14]. Mechanistically, empagliflozin promoted a shift toward anti‐inflammatory M2 macrophages, reduced proinflammatory M1 macrophage infiltration, and interfered with the signal transducer and activator of the transcription 3 (STAT3) signaling pathway [14, 25]. Intriguingly, SGLT2 expression increased during the induction of inflammatory cytokines such as IL‐1β [14] and, conversely, the beneficial effect of empagliflozin on IL‐1β, along with other inflammatory markers, was associated with a significant reduction in SGLT2 expression, suggesting that SGLT2 may be associated with inflammation and may contribute to the cardioprotective effect of empagliflozin [14]. While the study did not primarily focus on the direct impact of empagliflozin on the NLRP3 inflammasome, the ability of empagliflozin to suppress IL‐1β and inflammation highlights its therapeutic potential in managing myocarditis [14]. Overall, these findings strongly support empagliflozin as a promising therapeutic option for autoimmune myocarditis, demonstrating its ability to reduce inflammation, modulate macrophage polarization, prevent fibrosis, and protect against myocardial damage beyond its glucose‐lowering effects.

Canagliflozin, another SGLT2 inhibitor, has also been shown to provide significant cardioprotective effects in EAM [15]. EAM mice were treated with different oral doses (20, 30, and 60 mg/kg) of canagliflozin [15]. 60 mg/kg canagliflozin significantly reduced the severity of cardiac damage in EAM mice, as evidenced by an improvement in cardiac pathological score, the heart weight/body weight (HW/BW) ratio, and a reduction in cTnT levels [15]. Of interest, canagliflozin reduced cardiac inflammation and improved cardiac function by downregulating NLRP3 inflammasome components, including NLRP3, ASC, and caspase‐1, as well as downstream inflammatory cytokines IL‐1β and IL‐18 [15]. Additionally, the effect of canagliflozin on the NLRP3 inflammasome was associated with a reduction in Th17 cell infiltration into heart tissue, confirming the beneficial effect of canagliflozin on EAM‐induced myocarditis [15]. Furthermore, canagliflozin reduced apoptosis markers such as the Bax/Bcl‐2 ratio and cleaved caspase‐3 levels, therefore preventing apoptosis in cardiomyocytes in the EAM group [15]. Overall, these findings highlight canagliflozin as a potent therapeutic agent for autoimmune myocarditis through the suppression of NLRP3 inflammasome activation.

In addition to SGLT2 inhibitors, sacubitril/valsartan (Sac/Val), an angiotensin receptor–neprilysin inhibitor (ARNI), has shown therapeutic effects in EAM mice treated with Sac/Val at an oral dose of 20 mg/kg/day [16]. Sac/Val significantly reduced Th17 cell populations in the myocardial tissue of EAM mice [16]. Furthermore, Sac/Val downregulated cardiac levels of RORγt, a transcription factor controlling differentiation of Th17 cells, in EAM mice [16]. Mechanistically, the anti‐inflammatory effect of Sac/Val has been attributed to upregulation of sGC‐α1 and sGC‐β1 along with downregulation of phosphorylated NF‐κB p65 in myocardial tissue [16]. However, Sac/Val had no effect on the expression of NLRP3, ASC, caspase‐1, IL‐1β, and IL‐18 in EAM mice. These findings suggest that the anti‐inflammatory effects of Sac/Val appear to be independent of the NLRP3 inflammasome [16].

### 2.3. Pharmaceutical and Other Treatments for ICI Myocarditis by Targeting the NLRP3 Inflammasome

Recent studies have provided significant insights into the role of the NLRP3 inflammasome in ICI‐related myocarditis and the potential therapeutic benefits of various compounds targeting this pathway [11, 18]. ICIs, including ipilimumab, have revolutionized cancer treatment by enhancing the host immune system response to tumors [18, 26]. However, these treatments can induce severe immune‐related adverse events (irAEs), such as myocarditis [18, 26]. The pathogenesis of ICI‐induced myocarditis is increasingly linked to the activation of the NLRP3 inflammasome [27]. Thus, targeting inflammasomes might be a useful treatment for ICI‐induced myocarditis.

Crocin, a bioactive compound found in saffron, has been shown to protect against ICI‐related myocarditis by targeting the NLRP3 inflammasome [17]. A study on the effects of crocin in a mouse model of ICI‐induced myocarditis showed that crocin significantly reduced myocardial inflammation, fibrosis, and injury [17]. In this study, mice were immunized with murine cTnI peptide to induce myocarditis, followed by the administration of anti‐mouse PD‐1 to mimic the effects of ICIs [17]. Crocin was administered at two doses, 10 and 50 mg/kg/day, to evaluate its cardioprotective effects [17]. Echocardiography showed that the higher dose of crocin improved LV function [17]. Furthermore, histopathological staining of cardiac tissues demonstrated reduced inflammatory infiltration and scar formation in both crocin‐treated groups [17]. Mechanistically, the cardioprotective effect of crocin was mediated by inhibiting activation of the NLRP3 inflammasome, as evidenced by the decreased expression of NLRP3, cleaved caspase‐1, and proinflammatory cytokines IL‐1β and IL‐18 following treatment with both doses of crocin [17]. These effects were associated with suppression of the NF‐κB signaling pathway, a key regulator of NLRP3 activation [17]. Overall, by mitigating NLRP3 in cardiomyocytes, crocin restored cardiac function and reduced inflammation in a mouse model of ICI‐induced myocarditis.

A recent study demonstrated that high glucose levels increased the activation of the NLRP3 inflammasome and worsened ICI‐induced cardiotoxicity in patients with breast cancer [18]. In this study, in contrast to low glucose conditions, breast cancer cells produced more leukotrienes and cytokines after being incubated with ipilimumab under conditions of hyperglycemia [18]. However, this effect was significantly reduced when cells were switched from high to low glucose environments with empagliflozin [18]. Similar results were obtained when the same treatments were applied to cardiomyocytes [18]. Notably, empagliflozin mitigated these adverse effects by reducing NLRP3 and MyD88 activation. Overall, this study is consistent with a role for the NLRP3 inflammasome in ICI‐induced myocarditis, particularly during hyperglycemic conditions, and highlights the potential protective effect of empagliflozin in ICI‐induced myocarditis.

## 3. Barriers to Clinical Translation

The preceding discussion highlights that inflammasome‐targeting therapies, particularly naturally derived NLRP3 inhibitors, have preclinical potential as therapies for myocarditis, reducing cardiac damage, and preserving heart function, as shown in animal models of myocarditis [11–13, 15–18, 25]. However, only a few preclinical studies have successfully clinically translated, due to several barriers [6, 28–31]. Pharmaceutical agents such as anakinra, canakinumab, IFN‐α, and IFN‐β advanced to early phase trials but then encountered challenges related to safety, efficacy, and trial design. Natural compounds, which normally exhibit better safety profiles than pharmaceuticals, remain understudied. This section examines the barriers preventing clinical transition of NLRP3 inflammasome‐targeting therapies, with a focus on why naturally derived inhibitors may offer a viable alternative. We also propose solutions to accelerate this implementation.

### 3.1. Challenges in Current Clinical Trials

Early clinical trials explored the potential of immunomodulatory agents like interferons (IFN‐α and IFN‐β) to indirectly suppress NLRP3 activation by eliminating viral triggers and dampening inflammation [29, 30]. These cytokines activate IFN‐stimulated genes (ISGs), amplifying antiviral responses in infected and bystander cells [6]. By combining viral clearance with immunomodulation, these trials laid the foundation for targeted strategies to improve cardiac outcomes in both viral and immune‐mediated myocarditis.

Kühl et al. investigated the use of IFN‐β to target the NLRP3 inflammasome in patients with chronic viral myocarditis and LV dysfunction [29]. The trial demonstrated that 24 weeks of IFN‐β administration successfully eliminated enteroviral and adenoviral genomes in all 22 patients, accompanied by significant improvements in LV ejection fraction and reductions in LV diameters [29]. The treatment was well tolerated, with no serious adverse effects, suggesting that antiviral therapy could reverse myocardial dysfunction in virus‐positive patients [29].

Similarly, Ref. [30] tested IFN‐α to target the NLRP3 inflammasome in patients with myocarditis or idiopathic IDC. This open‐label trial found that immunomodulatory therapy improved LV function and physical endurance compared to conventional treatment alone [30]. The results from the trial revealed enhanced NK cell activity and potential viral clearance as outcomes [30]. However, the study lacked placebo controls and virological confirmation, therefore limiting definitive conclusions [30].

While these studies [29, 30] demonstrated the potential of interferon therapy in patients with viral myocarditis, clinical trials specifically targeting the NLRP3 inflammasome in myocarditis have faced significant challenges, often leading to inconclusive or negative results.

Recruitment issues continue to challenge clinical studies. Myocarditis is a heterogeneous disease with various etiologies, as described above, making it difficult to enroll a uniform patient population [6, 23, 30, 32]. Some trials have failed to stratify patients based on underlying pathophysiology, such as viral persistence vs. autoimmune‐driven inflammation, leading to mixed responses [30]. For example, in Ref. [29], the researchers specifically selected patients with PCR‐proven enteroviral/adenoviral persistence, which likely contributed to their success. Trials utilizing IFN therapies without strict virological inclusion criteria might include nonresponders, as interferons specifically target viral replication and antiviral immune responses, making it ineffective in nonviral or autoimmune‐driven myocarditis. This context is reflected in the trial performed by Ref. [30], in which the inclusion criteria did not require virological confirmation, leading to a mixed study cohort. The broader inclusion criteria diluted the results, as patient responses might have been more common in those with viral myocarditis and nonresponses in those with autoimmune or ICI myocarditis, where IFN is ineffective.

Furthermore, flaws in trial design may have also contributed to failures. For example, the sample size in the trial described in Ref. [30] was small and the trial was underpowered, probably because myocarditis is relatively rare compared with other cardiovascular conditions [33]. The underpowering of the trial may have obscured true treatment effects, emphasizing the need for larger studies that achieve adequate statistical power.

High dropout rates further complicate these trials, particularly in studies involving immunomodulatory or immunosuppressive therapies. In more severe cases of myocarditis, where cardiac function may already be compromised, the immunosuppressive effects of certain treatments can significantly increase infection risks, leading to adverse events that cause early discontinuation [34]. For example, therapies targeting broad immune suppression can impair innate and adaptive immune defenses, leaving patients vulnerable to bacterial, viral, or opportunistic infections [30]. This risk is especially notable in individuals with pre‐existing comorbidities or those receiving concurrent immunosuppressive medications (e.g., ICIs in cancer patients) [34].

The trial described in Ref. [30] reported that flu‐like symptoms, including fever, fatigue, muscle aches, and chills, were common side effects of IFN‐α therapy. These adverse effects likely contributed to treatment discontinuation in some participants. Although the researchers noted that these symptoms were typically transient and could be managed with supportive care like NSAIDs, the acute onset and severity of these reactions, often appearing within hours of injection, may not have been tolerated by some patients. This was particularly noticeable in those already compromised by heart failure. The characteristic “flu syndrome” associated with interferon therapy might have led some participants to withdraw from the study rather than enduring repeated cycles of these side effects over the 3‐month treatment period [35].

Safety and toxicity concerns remain a major hurdle in the clinical application of inflammasome inhibitors, particularly those targeting IL‐1β and IL‐18. Suppressing IL‐1β and IL‐18 can lead to immunosuppression, increasing susceptibility to infections [36]. For example, the Three C Study, where canakinumab was administered to patients with COVID‐19 with myocardial injury, noted concerns about bacterial infections, given the drug’s mechanism of action [31]. Similarly, in the ARAMIS trial, anakinra’s use in acute myocarditis required careful monitoring for infections, as IL‐1β plays a critical role in host defense against pathogens [28].

Second, off‐target effects may occur due to cytokine blockade and disrupted immune homeostasis. This can potentially exacerbate inflammation in unintended tissues or trigger organ toxicity. For instance, IL‐1β inhibition is associated with elevated liver enzymes and hematologic abnormalities, as seen in a previous trial of canakinumab for cardiovascular disease [9].

The CANTOS trial illustrates these key challenges [9]. This phase III study investigated IL‐1β inhibition with canakinumab for reducing cardiovascular disease in high‐risk atherosclerosis patients with persistent inflammation [9]. While this trial demonstrated that canakinumab reduced recurrent cardiovascular events, it also showed a dose‐dependent increase in fatal infections. This highlights the challenging balance between anti‐inflammatory benefits and immunosuppressive risks when administering inflammasome inhibitors [9].

Similarly, in the Three C Study, the benefits of IL‐1β inhibition were overshadowed by safety concerns, potentially contributing to the limited progression of these therapies into later‐phase trials [31]. The study population, which included elderly patients with multiple comorbidities, were more vulnerable to infection‐related complications, which is a known risk of IL‐1β blockade [31]. Furthermore, the requirement for elevated CRP levels (> 50 mg/L) as an inclusion criterion identified patients with severe systemic inflammation, in which immunosuppressive therapy could potentially worsen outcomes [31]. These safety concerns, combined with the observed adverse treatment effects, likely contributed to the study’s limited impact on clinical practice.

While short‐term studies of NLRP3 inflammasome inhibition in myocarditis have demonstrated promising reductions in some inflammation markers, their ability to prevent long‐term complications, such as myocardial fibrosis or progression to DCM, remains unclear [28, 31]. The ARAMIS trial [28] showed that the IL‐1 receptor antagonist anakinra improved short‐term outcomes (e.g., days free from myocarditis complications), but it did not assess long‐term cardiac remodeling or fibrosis regression. Similarly, the Three C study [31] found that canakinumab reduced acute inflammation in COVID‐19–related cardiac injury, but its impact on preventing chronic myocardial dysfunction was not evaluated. While NLRP3 inhibitors show promise for acute inflammation, their long‐term benefits in preventing myocarditis complications remain uncertain.

A further concern is tachyphylaxis, where NLRP3 inhibition may lead to the activation of other inflammatory pathways, diminishing therapeutic efficacy over time [37]. For instance, in the ARAMIS trial, the effects of anakinra were assessed only during the acute phase (28 days), leaving its long‐term efficacy for preventing myocarditis‐induced heart remodeling unexplored [28]. These findings highlight the need for longer‐term studies to determine whether NLRP3 inhibition can provide durable protection against adverse cardiac remodeling or if compensatory inflammation limits the therapeutic potential in chronic myocarditis treatment.

Moreover, there are no validated biomarkers to guide treatment responses. Both the ARAMIS and Three C trials incorporated biomarkers such as CRP or troponin for patient selection or endpoint assessment. However, neither addressed the need for the use of biomarkers to predict long‐term treatment responses or detect compensatory inflammation [28, 31]. Therefore, addressing this gap would help improve the reliability of clinical trial outcomes and facilitate the development of targeted therapies.

### 3.2. Solutions for Clinical Translation

Targeting NLRP3 in myocarditis shows promise but faces challenges such as disease heterogeneity and safety concerns. Therefore, some future solutions include optimizing clinical trial designs, addressing safety concerns, and exploring the roles of emerging technologies, thereby advancing NLRP3 inflammasome inhibition into an effective treatment for myocarditis.

Specifically, optimizing clinical trial designs—such as through better patient selection, application of combination therapies, and adaptive trial designs—offers a promising solution for clinical translation of NLRP3 inflammasome‐targeting therapies. By implementing these solutions, the hope is that response rates will improve (as only those patients more likely to respond are selected, i.e., personalizing the approach) and side effects will decrease. Targeted patient selection in clinical trials is critical for demonstrating the efficacy of NLRP3 inhibitors in myocarditis. Current trials often include heterogeneous populations, which may obscure treatment benefits, as reflected in [30]. Currently, there are no specific markers for NLRP3 activation in myocarditis, an issue that requires further research to address. However, myocarditis is one systemic manifestation of rheumatoid arthritis (RA), where both conditions activate the NLRP3 inflammasome [38, 39]. Specifically, studies of RA have identified key biomarkers of NLRP3 activation (ASC, caspase‐1, cleaved GSMD, IL‐1β, and IL‐18), and these could potentially be implemented in myocarditis [40, 41]. Future studies should validate these biomarkers of inflammasome activation to identify patients with active NLRP3‐driven inflammation for better patient selection in clinical trials.

Combination therapies represent another promising approach for improving myocarditis outcomes. NLRP3 inhibitors could be paired with standard heart failure treatments like SGLT2 inhibitors, which have shown anti‐inflammatory properties in preclinical models [15]. These combinations may simultaneously target multiple pathological pathways while potentially allowing for lower doses of individual drugs, thereby reducing side effects.

Furthermore, adaptive trial designs could help overcome some of the challenges posed by myocarditis heterogeneity [42]. These flexible protocols allow for modifications based on preliminary results, such as adjusting dosages or adding new treatment arms for nonresponders. This approach may be particularly valuable given the variable clinical course of myocarditis and the need to balance efficacy with safety concerns.

Addressing safety concerns should also be a priority in NLRP3‐targeted therapy development, particularly given the risks of broad immunosuppression [30, 34]. The development of more selective inflammasome inhibitors could significantly improve the safety profile of these therapies. Current IL‐1β blockers affect multiple inflammatory pathways, increasing infection risks [9, 30, 36]. Future compounds that specifically target NLRP3 components without broadly suppressing immunity are required, which may offer better risk–benefit profiles.

Similarly, a personalized medicine approach could further enhance safety by identifying patients most likely to benefit. Genetic testing for specific markers, such as NLRP3 pathway variants, and detailed immune profiling before treatment initiation might help predict both therapeutic response and susceptibility to adverse effects [43]. This strategy would allow clinicians to tailor treatments to individual patients’ needs.

### 3.3. Emerging Therapies for NLRP3 Inflammasome Modulation in Myocarditis

Recent advances in molecular therapeutics offer new therapeutic approaches to NLRP3 inflammasome modulation in myocarditis [44]. Among these approaches, RNA‐based therapies represent a novel method to modulate inflammasome activity with potentially greater specificity than current options. Specifically, RNA interference (RNAi) strategies, such as siRNA or anti‐miRs, could be employed to silence NLRP3 or its upstream regulators [44]. For example, the siRNA drug inclisiran, which targets PCSK9, showcases the potential of RNAi for cardiovascular applications [44]. These approaches could be adapted to myocarditis by designing siRNAs against NLRP3 components.

Furthermore, gene editing technologies like CRISPR offer the possibility of NLRP3 modulation [45]. This modulation can potentially provide long‐term protection against recurrent inflammation. Preclinical studies show that CRISPR‐Cas9‐mediated disruption of NLRP3 reduces inflammatory cytokine release in autoinflammatory models [45]. While this finding supports the potential of CRISPR‐based NLRP3 editing for potential anti‐inflammatory therapy, further research is needed to optimize delivery systems and ensure clinical safety before translation to myocarditis treatment.

Therefore, there is significant potential for NLRP3 inflammasome‐targeting therapies for myocarditis. Nevertheless, significant translational barriers must be overcome to realize their clinical benefits. To bridge the gap between preclinical studies and clinical application, rigorous trial designs, such as incorporating biomarker‐driven patient selection, adaptive protocols, and long‐term outcome assessments, are essential. Safety optimization, particularly for synthetic inhibitors, and increased investment from the pharmaceutical industry are equally important. Without addressing these challenges, these therapies risk remaining confined to experimental studies. Notably, natural products such as colchicine, calpastatin, morroniside, empagliflozin, canagliflozin, sacubitril/valsartan, and crocin, highlighted in this review, offer promising alternatives with potentially favorable safety profiles, requiring further exploration. A systematic and multidisciplinary approach is necessary to translate inflammasome‐targeting therapies from preclinical potential into clinically viable treatments for myocarditis.

## 4. General Overview and Conclusions

Myocarditis remains a significant global health concern, with increasing incidence and complex etiology that includes viral infections, autoimmune disorders, and ICI‐induced toxicity [3]. The disease contributes significantly to cardiovascular morbidity and mortality, often through heart failure, DCM, and sudden cardiac death [1, 3]. Inflammation‐induced cardiac remodeling is one of the most significant effects of myocarditis, characterized by excessive fibrosis, necrosis, and myocardial apoptosis [10]. These changes disrupt the structural integrity of the heart and impair contractility. However, despite advances in understanding the pathogenesis of myocarditis, these have yet to be translated to new treatments in the clinic.

Recent studies suggest that inflammasomes, particularly the NLRP3 inflammasome, are a key mediator of the damaging inflammatory response in myocarditis [4–6]. The NLRP3 inflammasome has emerged as a crucial member of myocarditis, linking pathogen‐associated molecular patterns (PAMPs) and DAMPs to inflammatory responses that contribute to myocardial injury [7]. When activated, it triggers the release of proinflammatory cytokines such as IL‐1β and IL‐18, leading to immune cell recruitment, cardiac remodeling, fibrosis, and eventual myocardial dysfunction [12–18]. The persistent activation of inflammasomes contributes to the progression of myocarditis into chronic inflammatory cardiomyopathy. Considering the detrimental effects of prolonged inflammation on the myocardium, targeting the NLRP3 inflammasome offers a potential therapeutic strategy to mitigate these effects and preserve cardiac function.

There is now abundant evidence on the use of different pharmaceutical and other treatments to target the NLRP3 inflammasome in viral, autoimmune, and ICI‐induced myocarditis. For instance, in viral myocarditis, medicines such as colchicine and calpain inhibitors have demonstrated the ability to suppress inflammasome activation, reduce pyroptosis, and prevent fibrotic remodeling [11, 12]. Similarly, natural factors and products such as morroniside have shown promise in limiting inflammatory damage and promoting myocardial repair [13]. Interestingly, SGLT2 inhibitors like empagliflozin and canagliflozin have been found to reduce inflammation by modulating macrophage polarization and inhibiting inflammasome activation in autoimmune myocarditis as well as ICI‐induced myocarditis [14, 15, 17, 18]. These data are consistent with previous studies, demonstrating that SGLT2 inhibitors improve cardiac function by targeting the NLRP3 inflammasome [24]. Unlike the aforementioned studies, sacubitril/valsartan has shown therapeutic potential in mitigating immune‐mediated damage through Th17 cell suppression and NF‐κB pathway inhibition, independent of NLRP3 inflammasome activation [16]. While further studies are needed to explore the association between the NLRP3 inflammasome and the adaptive immune response, the effect of sacubitril/valsartan on EAM might be due to a direct impact on the downstream adaptive immune response.

However, the clinical translation of NLRP3‐targeted therapies for myocarditis faces significant challenges, including patient heterogeneity, suboptimal trial designs (e.g., underpowered studies or inappropriate endpoints), and safety concerns related to immunosuppression, particularly with IL‐1β inhibitors like anakinra, which carry infection risks. While interferons (IFN‐α/β) have demonstrated efficacy in viral myocarditis, their utility in nonviral subtypes has yet to be established, highlighting a need for etiology‐specific strategies. In addition, emerging approaches such as RNAi or CRISPR require further refinement in delivery and safety. Given these limitations, natural products represent a promising alternative due to their favorable safety profiles; however, rigorous clinical evaluation is essential to establish their therapeutic efficacy and mechanisms of action.

In this review, we highlight the promise of NLRP3 inflammasome‐targeting therapies in managing myocarditis across different etiologies through various natural and pharmaceutical treatments. While these interventions show significant potential in preclinical models, challenges remain in turning these findings into clinical applications. Limitations include the need for large‐scale human trials, long‐term safety assessments, and the identification of optimal doses and treatment windows. Future research should focus on refining these therapeutic strategies, exploring other inflammasome inhibitors, and targeting other pathways to treat myocarditis.

**Nomenclature**
AIM2: association with absent in melanoma 2
ARNI: angiotensin receptor–neprilysin inhibitor
ASC: apoptosis‐associated speck‐like protein containing a CARD
CANTOS: Canakinumab Anti‐Inflammatory Thrombosis Outcomes Study
CARD: caspase‐recruitment domain
CRISPR: clustered regularly interspaced short palindromic repeats
CRP: C‐reactive protein
cTnI: cardiac troponin I
CVB3: coxsackievirus B3
DAMPs: damage‐associated molecular patterns
DCM: dilated cardiomyopathy
EAM: autoimmune myocarditis
HW/BW: heart weight/body weight ratio
IL: interleukin
irAEs: immune‐related adverse events
ISGs: IFN‐stimulated genes
LV: left ventricular
NLRP3: nucleotide‐binding domain leucine‐rich repeat pyrin domain‐containing 3
PAMPs: pattern‐associated molecular patterns
PRRs: pattern recognition receptors
RA: rheumatoid arthritis
RNAi: RNA interference
Sac/Val: sacubitril/valsartan
SGLT2: sodium/glucose cotransporter 2
SLE: systemic lupus erythematosus
STAT3: signal transducer and activator of the transcription 3

## Author Contributions

Faizah D. Retnowati and Dunia R. Halawa reviewed, collected the data, and wrote the first draft. Zaid H. Maayah and Atiyeh M. Abdallah conceptualized the project, edited the manuscript, and raised the funds.

## Funding

This publication was supported by Qatar University, under Internal Grant No.: QUCG‐CHS‐25/26‐736.

## Disclosure

The findings achieved herein are solely the responsibility of the authors.

## Conflicts of Interest

The authors declare no conflicts of interest.

## Data Availability

Data sharing is not applicable to this article as no datasets were generated or analyzed during the current study.
